# Geospatial analysis of distribution of community pharmacies and other health care facilities providing minor ailments services in Malaysia

**DOI:** 10.1186/s40545-021-00308-9

**Published:** 2021-02-24

**Authors:** Mei Mei Tew, Ernieda Hatah, Faiz Arif, Muhammad Aqiff Abdul Wahid, Mohd Makmor-Bakry, Khairul Nizam Abdul Maulad

**Affiliations:** 1 Faculty of Pharmacy, Universiti Kebangsaan Malaysia, Jalan Raja Muda Abdul Aziz, 50300 Kuala Lumpur, Malaysia; 2 Clinical Research Centre, Hospital Sultan Abdul Halim, Sungai Petani, 08000 Kedah Malaysia; 3 Earth Observation Centre, Institute of Climate Change, Universiti Kebangsaan Malaysia, Bandar Baru Bangi, 43650 Selangor Malaysia; 4 Department of Civil Engineering, Faculty of Engineering & Built Management, Universiti Kebangsaan Malaysia, Bandar Baru Bangi, 43650 Selangor Malaysia

## Abstract

**Background:**

Minor ailments are defined as common, self-limiting, or uncomplicated conditions that may be diagnosed and managed without a medical intervention. Previous studies reported that pharmacists were able to help patients self-manage minor ailments that led to a reduction of health care burden in other facilities. Nevertheless, public access to community pharmacy and other health care facilities offering services for minor ailments has not yet been explored in Malaysia. Hence, this study aims to determine population access to the above-mentioned services.

**Method:**

According to the reported practice address in 2018, the spatial distribution of health care facilities was mapped and explored using the GIS mapping techniques. The density of health care facilities was analyzed using thematic maps with hot spot analysis. Population to facility ratio was calculated using the projection of the population growth based on 2010 census data, which was the latest available in the year of analysis.

**Results:**

The study included geographical mapping of 7051 general practitioner clinics (GPC), 3084 community pharmacies (CP), 139 public general hospitals (GHs) and 990 public primary health clinics (PHC). The health care facilities were found to be highly dense in urban areas than in the rural ones. There were six districts that had no CP, 2 had no GPC, and 11 did not have both. The overall ratio of GPC, CP, GH, and PHC to the population was 1:4228, 1:10,200, 1:223,619 and 1:31,397, respectively. Should the coverage for minor ailment services in public health care clinics be extended to community pharmacies, the ratio of facilities to population for each district would be better with 1:4000–8000.

**Conclusions:**

The distribution of health care facilities for minor ailment management in Malaysia is relatively good. However, if the scheme for minor ailments were available to community pharmacies, then the patients’ access to minor ailments services would be further improved.

## Backgroud

Minor ailments are uncomplicated, common, or self-limiting conditions, which could be managed through self-care. They usually do not require medical intervention or a doctor’s prescription [1]. Patients with minor ailments can seek advice and treatment from various health care professionals such as general practitioners, primary care medical practitioners, and community pharmacists. Nevertheless, the study has revealed that patients seek treatment for minor ailments also from the emergency department (ED) of hospitals. Since it is very common, minor ailments are reported to consume a significant number of doctors’ appointments and emergency department visits [2]. This has placed unnecessary strain on the health care facilities, the focus of which should be on chronic or emergency cases. For instance, in the United Kingdom (UK), about 5% of the ED visits and 13% of general practitioners appointments were due to minor ailments [3, 4]. Norway reported a higher percentage of emergency department attendance for minor ailments, which was 28% [5]. In Malaysia, an alarming number of inappropriate emergency department visits for non-critical cases have also been observed with 55% and 62.1% of such visits being for inappropriate and/or non-critical cases, respectively [6, 7]. The reasons, however, for utilizing the emergency department for minor ailment management are unknown.

Community pharmacists play an important role as a primary health care professional [8]. They are often an entry point to the health system from whom the public can seek consultations on a variety of issues such as lifestyle, drug and non-drug treatments, advice on symptoms and sales of products, and managing minor ailments without an appointment. In minor ailment management, the community pharmacists can support the customers’ wish for self-care and, if necessary, refer them to appropriate healthcare professionals. Internationally, many countries have promoted the role of pharmacists in managing minor ailments. This includes the introduction of the Minor Ailment Service (MAS) scheme that enables people with minor health conditions to access medicines and advice without visiting their doctor. The scheme also aims to encourage people to take responsibility of their own health, reduce the demand for more expensive healthcare options such as appointments with primary care medical practitioners or visits to hospital emergency departments, and promote the efficient use of scarce public resources [9]. The schemes were reported to be implemented in several countries such as in the UK, Saskatchewan province of Canada, New Zealand, Australia, Spain, and other European countries [10]. It were found to reduce GP workload, improve patients’ access to medicines, and provide patients with greater choices of primary health care services [11]. The level of satisfaction among the patients receiving the services of the scheme was found to be high, particularly due to ease of access and convenience [12].

In Malaysia, although a scheme for minor ailments has yet not been implemented, the practices of being self-medicated and getting advice from community pharmacists for minor ailments have been widely reported [13]. Community pharmacists are supportive of patients’ self-care for minor ailments through the use of over-the-counter medicine, or pharmacist-only medicines known as Poison C items that can be purchased from a pharmacist without a doctor's prescription. While good geographical access to community pharmacies might enable the public to utilize them for minor ailment management, a lack of such access could reduce the resultant public benefits [14]. A previous study by the Malaysia National Health and Morbidity Survey in 2015 reported that 43.1% and 47.7% of the population stays within 5 km of the government out-patient and private out-patient health care facilities, respectively [13]. The survey, however, did not examine the geographical distribution of community pharmacies or the population ratio to it.

There are few important components in defining access to healthcare. First and foremost, the geographical or physical accessibility. Good geographical access has been defined as the availability of good health services within reasonable reach of those who need them and of opening hours, appointment systems and other aspects of service organization and delivery that allow people to obtain the services when they need them. Second, acceptability and timeliness availability of the healthcare services, which captures people’s willingness to seek services. Last but not least, financial affordability of the health care services. Good geographical access to unaffordable health care services would be meaningless as people would not be able to utilize it. This will increase social inequities and push people into poverty when they have to pay for health services out of their own pockets. Hence, to ensure population’s health and welfare, geographical access to affordable health care services such as the Universal Health Coverage (UHC) need to be the hallmark of a government’s commitment to improve the well-being of all its citizens [15, 16].

Using geospatial analysis, which is a powerful tool to evaluate and analyze spatial data, the geographical distribution of health care services providing minor aiments will provide understanding whether there are any dominant patterns in the distribution of and access to healthcare facilities. With a view to expanding the impact of the distribution, the Average Nearest Neighbours is the best approach to determine and understand whether there are any dominant patterns in the distribution of healthcare facilities. This study, therefore, aims to quantify and compare the spatial distribution of community pharmacies, public primary health clinics, public general hospitals, and general practitioner clinics in Malaysia, and to study the ratio of population to the facilities for minor ailment-related services.

## Methodology

This cross-sectional study conducted the geomapping of all public and private health care facilities that provide treatment for minor ailments in Malaysia, such as community pharmacies, general practitioners’ clinics, public primary health clinics, and hospitals. The study evaluated all the 13 states and three Federal Territories in Malaysia that include West and East Malaysia located at 99°25′ and 119°33′ for the latitude, and 0°19′ and 7°52′ for the longitude, respectively.

The addresses of the registered community pharmacy facilities in Malaysia up to March 2018 were obtained from the License A registration list that is enlisted in the Pharmaceutical Services Program of the Ministry of Health (MOH) official portal [17]. Meanwhile, the database of addresses on general practitioner clinics and all MOH facilities that consisted of public primary health care clinics, known as ‘KlinikKesihatan’ and ‘Klinik 1 Malaysia’, and hospitals were obtained from the doctors’ list in the Malaysian Medical Council official portal with list updated up to October 2018 [18] and from the MOH directory [19]. In this study, public hospitals were also included because the previous studies reported the use of emergency departments for seeking minor ailment management by the public. The list of healthcare addresses then underwent substantial filtering to remove facilities that did not provide minor ailment treatments such as aesthetics, obstetrics and gynecology, eyes and pediatrics. These clinics were identified through the clinic’s name, website or through contacting of the facilities for confirmation. Community pharmacies that offer only wholesale business, identified through the type of license registration reported in the website, were also excluded from this study.

The addresses that were obtained in the format of number, street address, city, state, and postcode were then used to locate and identify the facilities using several mapping services such as Google Maps and street view, Here WeGo, and LatLong.net. Once the location of addresses was confirmed, it was then converted into World Geodetic System 84 geographical coordinates of latitude and longitude. The latitude and longitude data were then converted into shape file format using the Geographic Information System (ArGIS^©^) 10.5 software (ESRI Redlands, CA).

Average Nearest Neighbor was then performed to measure the distance between the locations of each feature centroid and its nearest neighbor's centroid. It then showed the average of all these nearest neighbor distances. If the average distance was less than that for a hypothetical random distribution, the distribution of the features being analyzed was considered as clustered and seen as a ‘hot spot’. Conversely, if the average distance was greater than that for a hypothetical random distribution, the features were considered as dispersed or ‘cold spots’. The average nearest neighbor ratio was calculated as the observed average distance divided by the expected average distance. Overlay analysis was conducted to assess the level of distribution, low and high, of all the included facilities. A 5 km buffer analysis that generate catchments at physical distances was done to investigate the area that had lack of access to any minor ailment services such as general practitioner’s clinic and/or community pharmacies [20]. The current study defines good geographical distribution of health care facilities based on two factors. First, 80% of the districts must have access to at least one public and one private health care facility in their areas. Second, good distribution is considered if number of health care facilities were proportionate according to population size in that area. This means that area with high population density should have more health care facilities than less dense area. The population ratio according to district estimation for 2018 was determined based on the 2010 Malaysia Population Census data and multiply it with the reported average population growth of 1.15% per year [21].

## Results

**Fig. 1:**
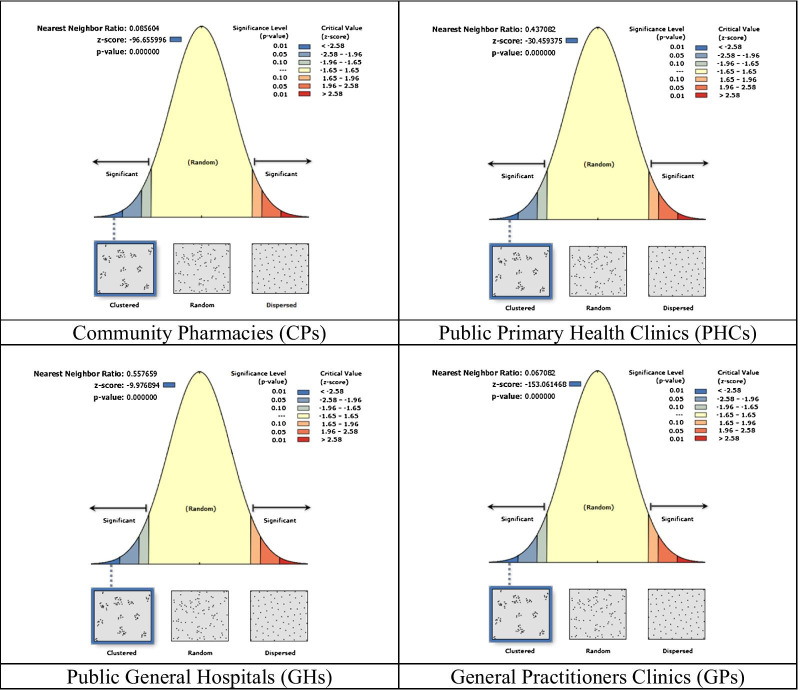
Spatial patterns of healthcare facilities in Malaysia

There was a total of 7051 general practitioner’s clinics, 3084 community pharmacies, 139 public hospitals, and 990 primary health clinic addresses that consisted of 790 ‘KlinikKesihatan’ and 191 ‘Klinik 1 Malaysia’—all of these were analyzed in this study. The summary of spatial pattern of healthcare facilities in Malaysia is presented in Fig. 1. The observed mean distance shows that the range of the distribution of the healthcare facilities is between 452.09 and 26,738.33 m. All types of healthcare facilities had the Nearest Neighbor Index of less than 1, which indicates that the distribution of clustered pattern is significant (*p*-value < 0.001). Pattern direction can be considered as dispersed if the index is more than 1.

**Fig. 2:**
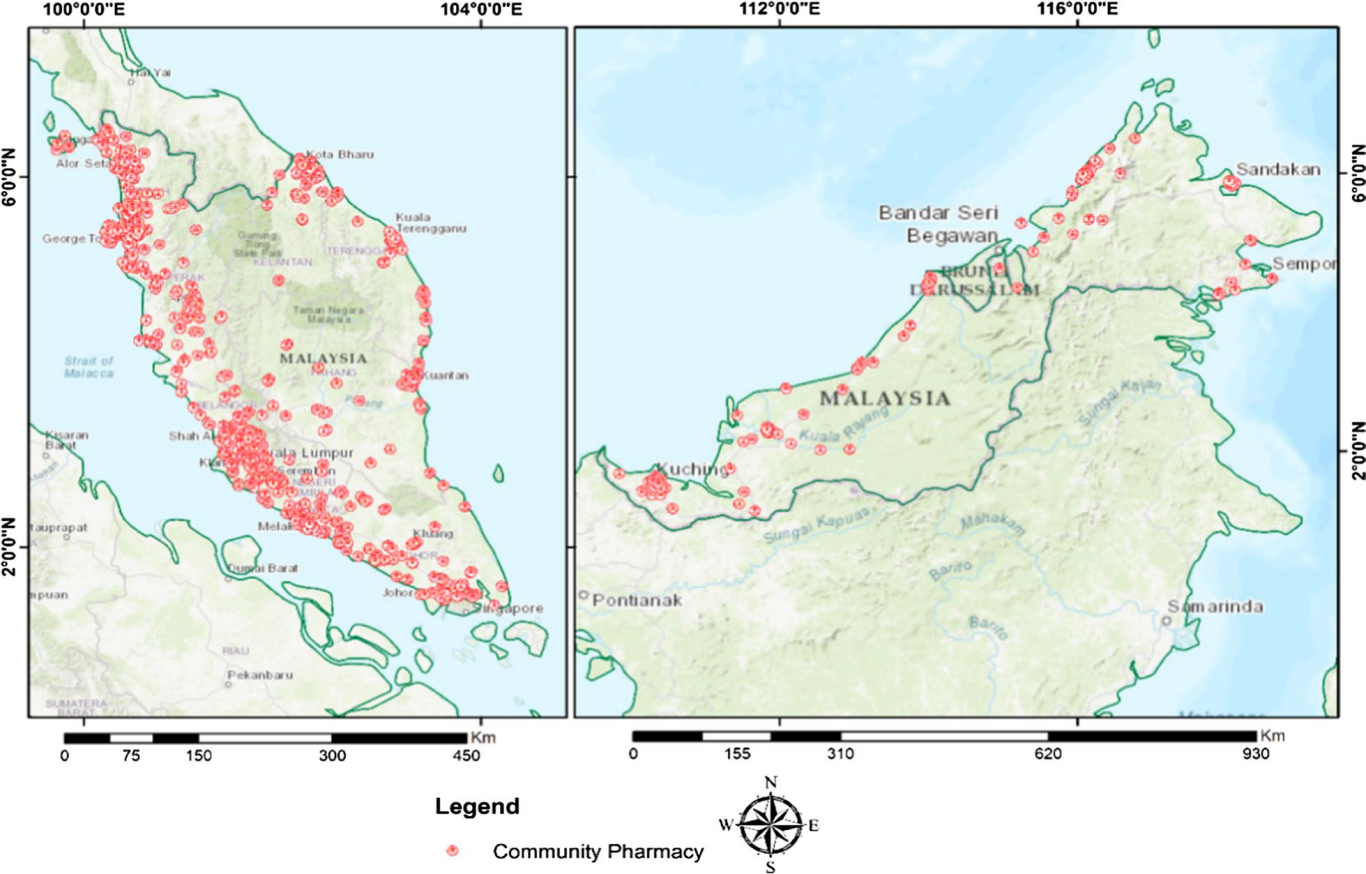
Community pharmacy locations in Malaysia by geographic regions, 2018

**Fig. 3:**
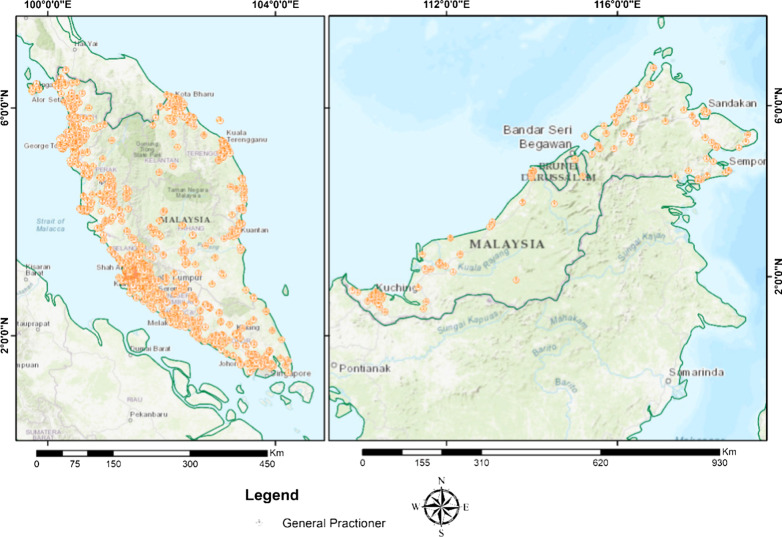
General practitioners clinics’ locations in Malaysia by geographic regions, 2018

**Fig. 4:**
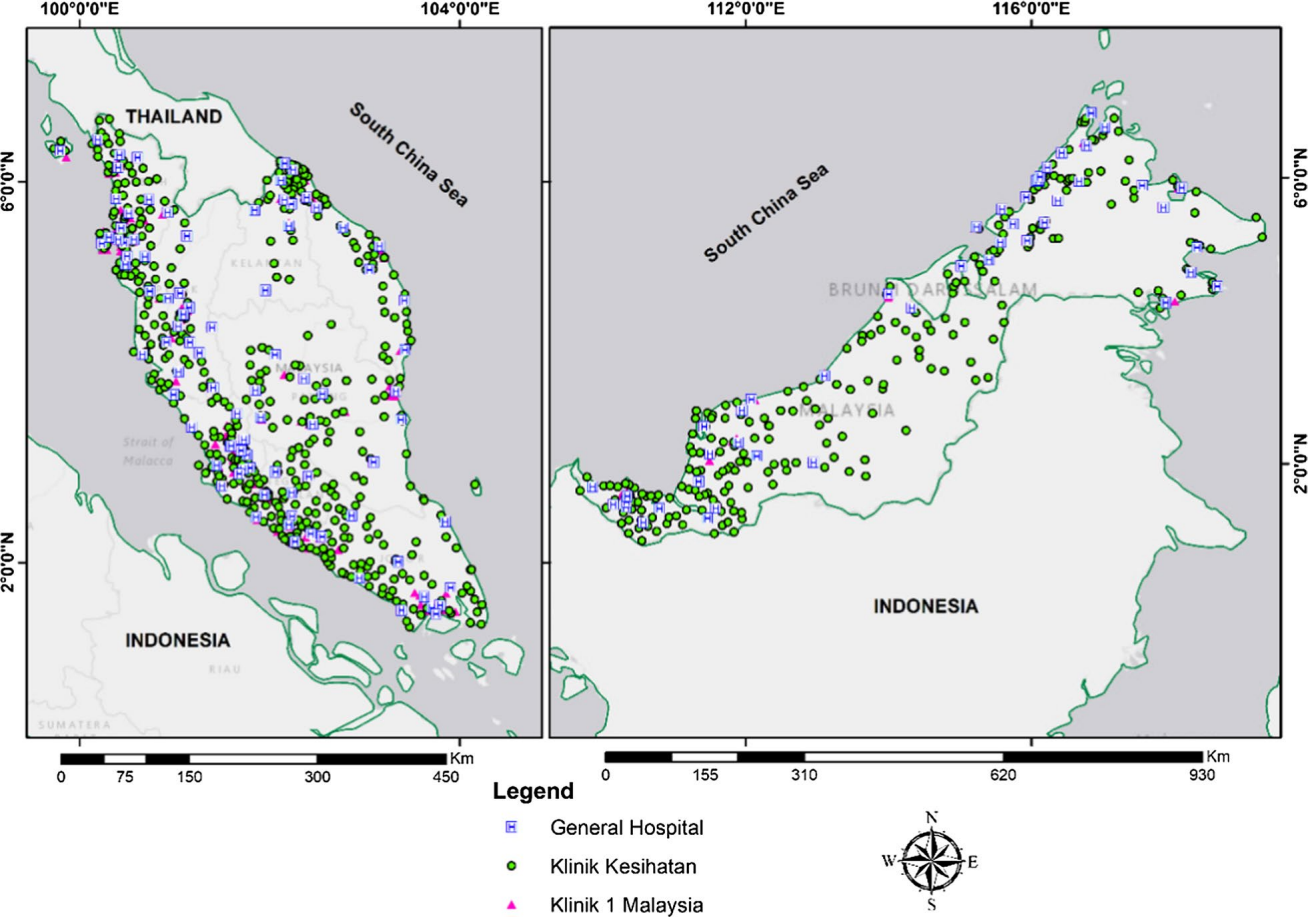
Public hospitals and primary health care clinics’ locations in Malaysia by geographic regions for 2018

**Fig. 5:**
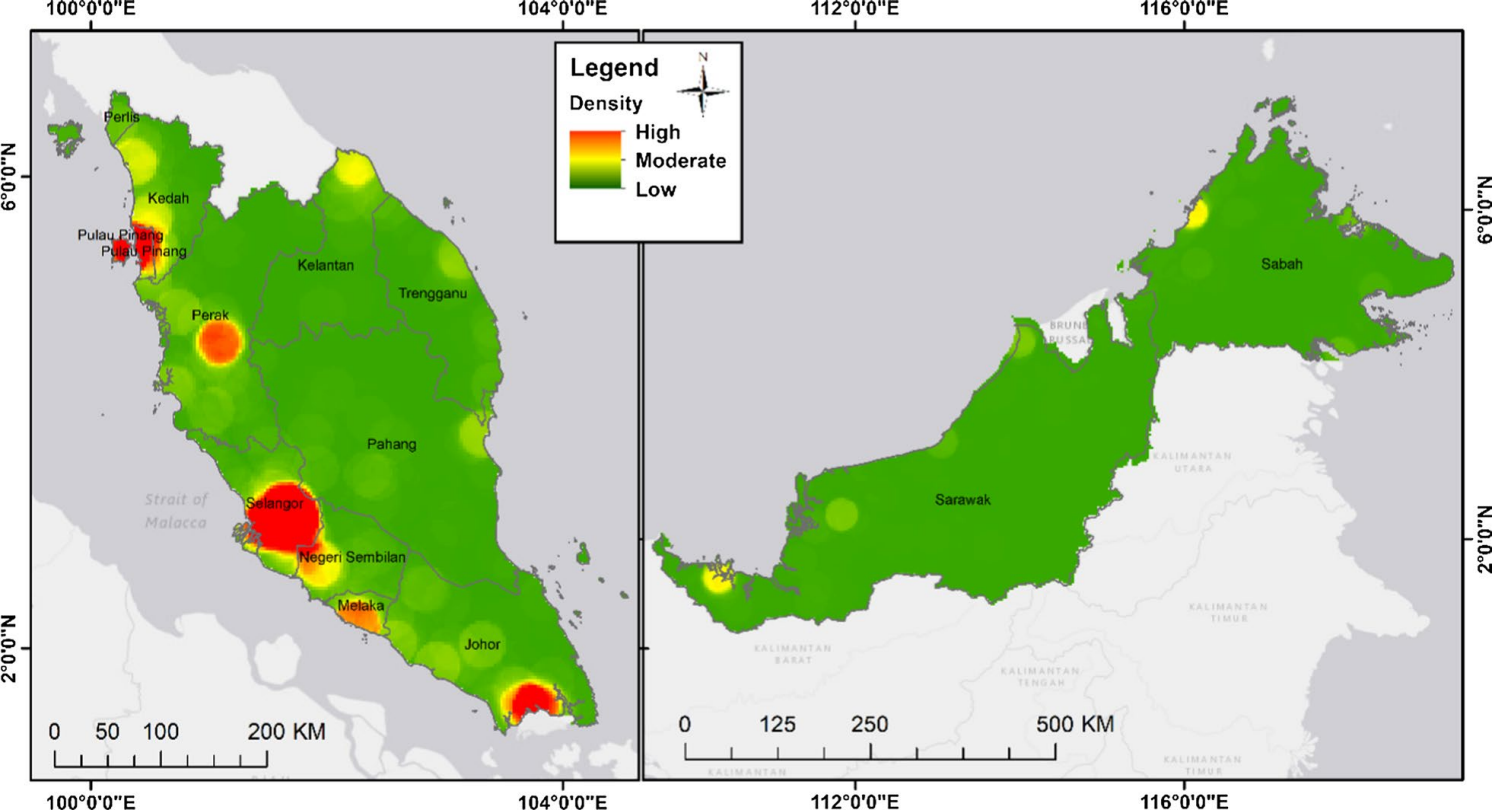
Thematic map with hot spot analysis for distribution density of all health care facilities (public and private) offering minor ailments services in Malaysia for 2018

The distribution of community pharmacies and general practice clinics as in March and December 2018 by geographic regions is shown in Figs. 2 and 3. The Malaysia population density according to district is provided in Additional file 1. The community pharmacies and general practice clinics are concentrated more in West Malaysia than in the East. A high density of general practice clinics and community pharmacies was seen in the urban area with high density population such as Pulau Pinang state in the north of West Malaysia, Selangor state in the central region of West Malaysia, and Johor Bahru district in Johor state in the southern region of West Malaysia. Ipoh district which is also an urban area located in the northern region of Malaysia has a moderate to high density of general practice clinics and community pharmacies. On the other hand, other states such as Kedah, Perak, Pahang, Kelantan, and Terengganu that with more suburban and rural areas with less dense population have moderate to low density distribution of general practice clinics and community pharmacies. In East Malaysia, a high number of general practice clinics and community pharmacies were seen in area with dense population such as Kuching and Miri in Serawak and Kota Kinabalu and Sandakan in Sabah. A rather good distribution of public healthcare facilities, hospitals, and primary care health clinics, was observed throughout Malaysia (Fig. 4). All districts were shown to have access to at least one public health care facility offering treatment for minor ailment. District with the lowest public health care facility is Penampang and Putatan in Sabah and Meradong in Serawak with only one public health clinic available each. The district with the highest number of public health care clinics is Marudi in Serawak with 24 health clinis available. West Malaysia has moderate to high density distribution of government healthcare facilities with the majority being concentrated in the states’ major cities such as Kota Bharu in Kelantan and Kuala Terengganu in Terengganu. The thematic map using hot spot analysis of all the health care facilities offering minor ailment services is shown in Fig. 5. In line with the population density (Additional file 1), Pulau Pinang, Selangor and Johor Bahru district in Johor which located in the urban area had the highest density of all types of healthcare facilities, followed by Ipoh and Melaka with moderate density of such distribution. In East Malaysia, moderate density of distribution of all health care facilities offering minor ailments services were seen only in Kuching and Kota Kinabalu districts which are the capital cities, located in the urban area, of Sabah and Serawak.

Through buffering analysis, it was noted that about 34 (1.12%) community pharmacies had no general practice clinics within their 5 km radius. Most of these community pharmacies were in Sarawak (*n* = 11, 32.4%) and Perak (*n* = 8, 23.5%). Meanwhile, a total of 237 (3.22%) general practice clinics had no community pharmacies within their 5 km radius, and these were mostly located in Sabah (*n* = 51, 21.52%), Johor (*n* = 37, 15.61%) and Selangor (*n* = 35, 14.77%). The overall ratio of community pharmacies to population in Malaysia is 1:10,200. The ratio of community pharmacies to population, according to districts, ranges between 1:4830 and 1:61,707 in West Malaysia, and between 1:6015 and 1:106,412 in East Malaysia. The lowest ratio of community pharmacies to population was in W.P Kuala Lumpur (1:4830), and the highest ratio was seen in the Sabah state with 1:20,032. The ratio of community pharmacies to general practice clinics, according to districts, was found to range between 1:0.8 and 1:7.0, and 1:0.3 and 1:8.3 for West and East Malaysia, respectively. Of the total of 144 districts in Malaysia, there were six districts without a community pharmacy, two without any general practice clinics, and 11 without both facilities. Example of districts that without both general practice clinic and community pharmacy were Nabawan and Tongod in Sabah and Belaga, Dalat and Julau in Serawak. This bring the percentage of districts without access to private health care services offering minor ailment treatment to only 7.6%. The summary of the type of healthcare facilities and its population ratio is presented in Table 1. The overall ratio of general practice clinics to population is 1:4228. There is a good ratio of general practice clinics to population, according to the state, with the lowest ratio being in W.P Kuala Lumpur (1:1548), and the highest ratio in Sabah (1:9633). The ratio of public hospitals to population was found to be generally higher, and it ranged between 1: 77,312 and 1:898,390; the public primary health care clinics to population ratio was found to range between 1:13,990 and 1:82,814.

**Table 1:** Ratio of healthcare facilities to population in each states in Malaysia (population estimates for year 2018)

States	Ratio GP vs population	Ratio CP vs population	GH vs population	PHC vs population
Overall	1:4228	1:10,200	1: 223,619	1:31,397
West Malaysia				
Perlis	1:6715	1:12,151	1:255,174	1:18,227
Kedah	1:6174	1:9766	1:238,723	1:35,221
Pulau pinang	1:3332	1:6440	1:287,640	1:37,518
Perak	1:4038	1:10,403	1:173,379	1:29,893
Selangor	1:3336	1:9581	1:503,782	1:82,814
Wp kuala lumpur	1:1548	1:4830	1:898,390	1:54,448
Wp putrajaya	1:3221	1:5522	1:77,312	1:38,656
Negeri sembilan	1:3941	1:8201	1:185,890	1:21,869
Melaka	1:3050	1:8351	1:297,865	1:24,151
Johor	1:4110	1:10,529	1:304,453	1:33,828
Kelantan	1:7425	1:18,688	1:184,808	1:25,201
Terengganu	1:6849	1:18,155	1:190,632	1:23,829
Pahang	1:7115	1:16,798	1:162,939	1:22,630
East Malaysia				
Sarawak	1:7936	1:10,770	1:123,367	1:13,990
Sabah	1:9633	1:20,032	1:146,900	1:36,725
Wp labuan	1:7301	1:18,982	1:94,908	1:47,454

*GP* general practitioners, *CP* community pharmacies, *GH* general hospitals, *PHC* public health clinics, *Wp* Wilayah Persekutuan

## Discussion

This study evaluated the distribution of healthcare facilities providing minor ailment treatment in Malaysia. The findings show that there was a slight uneven geographical distribution of private and public healthcare facilities offering treatment for minor ailments in Malaysia. Major cities such as Penang, Selangor, and Johor had a higher density of private health care facilities such as community pharmacies and general practice clinics than that in any other part of Malaysia. This is similar to the previously documented studies in other countries that reported private general practice clinics and community pharmacies to be more concentrated in urban areas than in rural ones [20, 22, 23]. There are many factors that may influence the uneven distribution of private healthcare facilities in urban and non-urban areas, and this includes the influence of population density, financial risk, earning potential, workload, and infrastructure [24, 25]. In addition, the distribution of health care facilities may also be influenced by public demand, which could be associated with education and health conscious level that is generally higher among the public in the urban areas. In accordance with the previously reported findings, higher education and income were reported to be associated with the use of private health care services [26, 27].

There were 11 districts in East Malaysia that were without access to either community pharmacy or general practice clinics. This could be due to low population in the areas that were between 15,000 and 40,000 people. Less health care facilities may affect public access to efficient medical care and medication therapy as reported in an Australia study. In the study, the public in the towns without community pharmacy were reported to have poorer access to prescription and non-prescription medications than those with community pharmacy [28]. Health services and health outcomes will suffer when access to health services or workers is scarce [29]. This was evident as high mortality rates for children under 5 years were seen in countries with low health workers to population ratios [30]. Since access to medication is a fundamental element of the right to health [31], it is important to encourage the setting up of community pharmacies and/or general practice clinics in these areas. This may include, for example, providing incentives to healthcare facilities to open their premises in areas where public have no access to medical care and medication, as practiced in New Zealand and Australia [32]. In New Zealand, ‘rural bonuses’ was provided as an incentive to improve the recruitment of GPs practicing in rural areas, and to offset the financial burdens of rural practice [33]. Using the Rural Ranking Scale (RRS), GPs in New Zealand were paid according to their score related to difficulties in delivering the services with higher scores attracting higher payments [34]. Incentivizing community pharmacies and GPs for providing treatment for minor ailments in rural areas may help in the following ways: providing the public with more choices related to primary health care treatment, overcoming the uneven distribution of healthcare facilities, improving access to medication, and reducing the over dependence on public healthcare infrastructure [35].

In the current study, there were six districts that without a community pharmacy within the 5 km radius of GPs settings. Since GPs in private health care settings in Malaysia could dispense their own prescriptions, they usually practiced in isolation without close collaboration with the community pharmacists. The coexistence of nearby general practice clinics and community pharmacy was seen as a competitive threat, especially in non-urban areas, where the population and demand for private primary healthcare services were generally lower. Absence of dispensing separation, lack of minor ailment treatment scheme, overlapping of services provided by the community pharmacies and GPs could be the reasons for the competitive environment among both the practices. A recent qualitative study in the UK revealed that the barrier for GP-pharmacist collaboration includes inter-professional tension arising from funding conflicts that cause service offers to be duplicated, causing inefficient workflow within the primary care pathway [36]. This did not benefit the public as it may have underutilized specific professional expertise, and fragmented the patient-care services.

The current study found that the overall population to community pharmacy ratio in Malaysia was between 1:9000, which is lower than the ratio reported in high income countries of 1:2000–8000 [37]. The International Pharmaceutical Federation suggests that the average population served by the community pharmacy be used as an indicator of a country’s community pharmacy infrastructure and capacity [38]. Only five states in Malaysia, namely, Pulau Pinang, W.P. Kuala Lumpur, W.P. Putrajaya, Negeri Sembilan, and Melaka had good community pharmacy to population ratio of 1:4000 to 8000, whereas the pharmacy to population ratio in other states ranged between 1:8000 and 18,000. The ratio was found to be higher, which is more than 1:10,000, especially in East Malaysia such as Sabah, Sarawak and Kelantan, Terengganu and Pahang located in the East Coast of West Malaysia. Higher community pharmacy to population ratio in these areas could be explained by geographical challenges such as mountainous terrain and sparse population [39]. In addition, the population in East Malaysia had about half of the median household income compared to those residing in Kuala Lumpur [40]. This could influence the distribution of private healthcare facilities in Malaysia. Similarly, geographical variation in the distribution of community pharmacies was also reported in the US with estimated community pharmacies per capita of 2.11 per 10,000 population. In some localities, fewer community pharmacies were found in the Southwest and Pacific west region of the country. Most of these regions reported a higher rate of non-adherence to medication, and the population encountered barriers in the availability of community pharmacies when they needed pharmacy services [37]. It is because of this that measures should be taken to encourage the equal and fair distribution of community pharmacies across all states in Malaysia to ensure good access to minor ailment services.

In the current study, the ratio of public hospital to population was found to range between 1:77,312 and 1:898,390, which is comparable to developed countries such as New Zealand [41]. Since the ratio of the population to public hospitals was higher than that found for community pharmacy and general practice clinics, the facilities were reported to be overburdened for non-serious cases such as minor ailments. Currently, despite minor ailments that can be treated in public primary health care clinics, the EDs were reported to be inappropriately used for non-emergency cases such as minor ailments [8, 9]. With good access and population to community pharmacy ratio, community pharmacy settings can be used as an alternative to reduce inappropriate visits to EDs. Should the government coverage of minor ailment treatment be extended to community pharmacies, the ratio of population covered per health care facility would be reduced to range between 4000 and 12,000 producing a better coverage for the public. With the extended coverage, majority of the states will achieve facilities to population ratio of 4000–8000. The population coverage of access to minor ailment management for critical areas in Sabah and W.P Labuan in East Malaysia as well as in Kelantan, Terengganu, and in Pahang states in the East Peninsular region would be improved to cater to a population range of 8000–12,000. However, this can only be achieved through minor ailment treatment scheme or with universal health coverage (UHC), which is currently not yet in place in Malaysia. Achieving UHC may ensure successful partnership between public and private health care sectors, and better utility of health care professionals and practices that can be a potential cost-saving factor for the government.

This study is limited by it cross-sectional nature in terms of study design. The mapping included only the distribution of the available health care facilities up to March and December 2018. In addition, the population to health care institution ratio in each district was mapped based on the census data that is not in geospatial vector data. Without the coordinate precision, the results on population to institution ratio may not be accurate. Nevertheless, the findings can be treated as an estimation of the population to health care institution ratio in Malaysia. Future studies may want to accurately estimate the population access to minor ailment management using the network analysis that includes evaluation of access of the routes that people can travel on such as roads.

## Conclusion

The distribution of healthcare facilities for minor ailment management in Malaysia is relatively good with all districts have access to at least one public and one private health care facilities, except in 11 districts which is without private health care facility offering minor ailment services. The density of health care facilities distribution was also found to be proportionately to the district population density. Nevertheless, health care facility to population ratio related to minor ailment treatment could be improved through minor ailment scheme or universal health coverage system that allow subsidized treatment to be obtained in private health care facilities such as general practices and community pharmacies.

## Supplementary Information

**Additional file 1**. Population density according to district in West and East Malaysia.

## Data Availability

The data sets during and/or analysed during the current study available from the corresponding author on reasonable request.
