# Supplementary figures and images for: Geospatial analysis of distribution of community pharmacies and other health care facilities providing minor ailments services in Malaysia
Source: J Pharm Policy Pract. 2021 Feb 24;14:24. doi: 10.1186/s40545-021-00308-9 (PMC7903721; doi:10.1186/s40545-021-00308-9)

**Additional file 1: Population density according to district in West and East Malaysia**


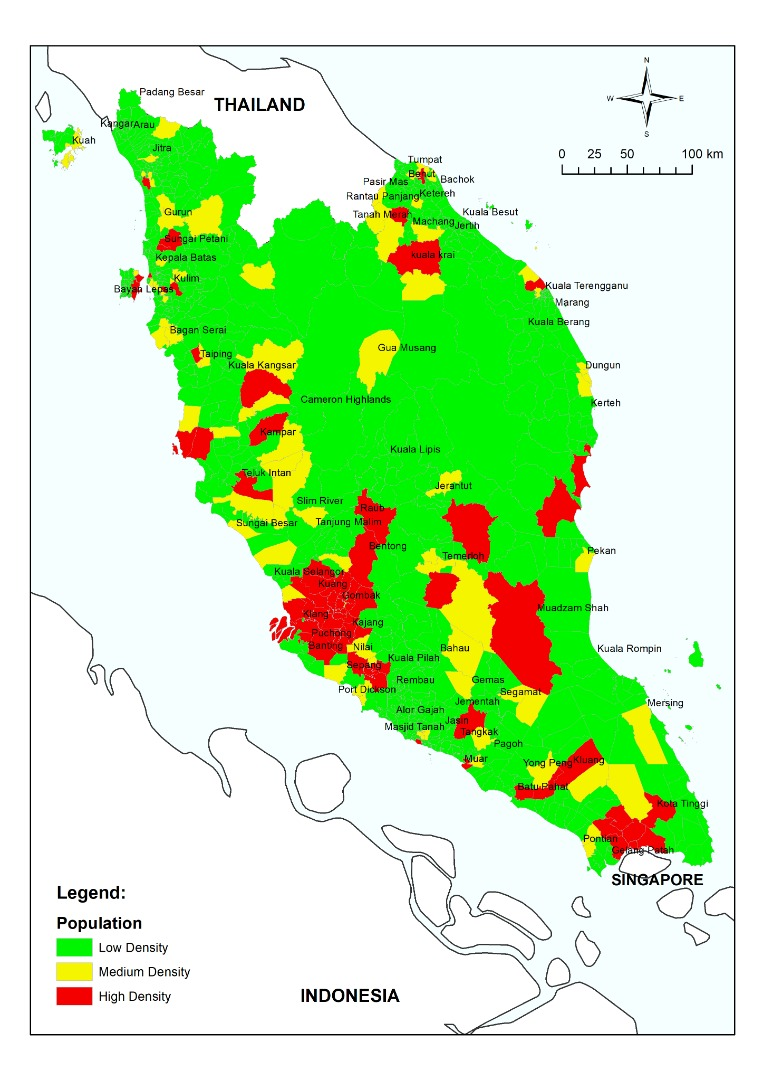


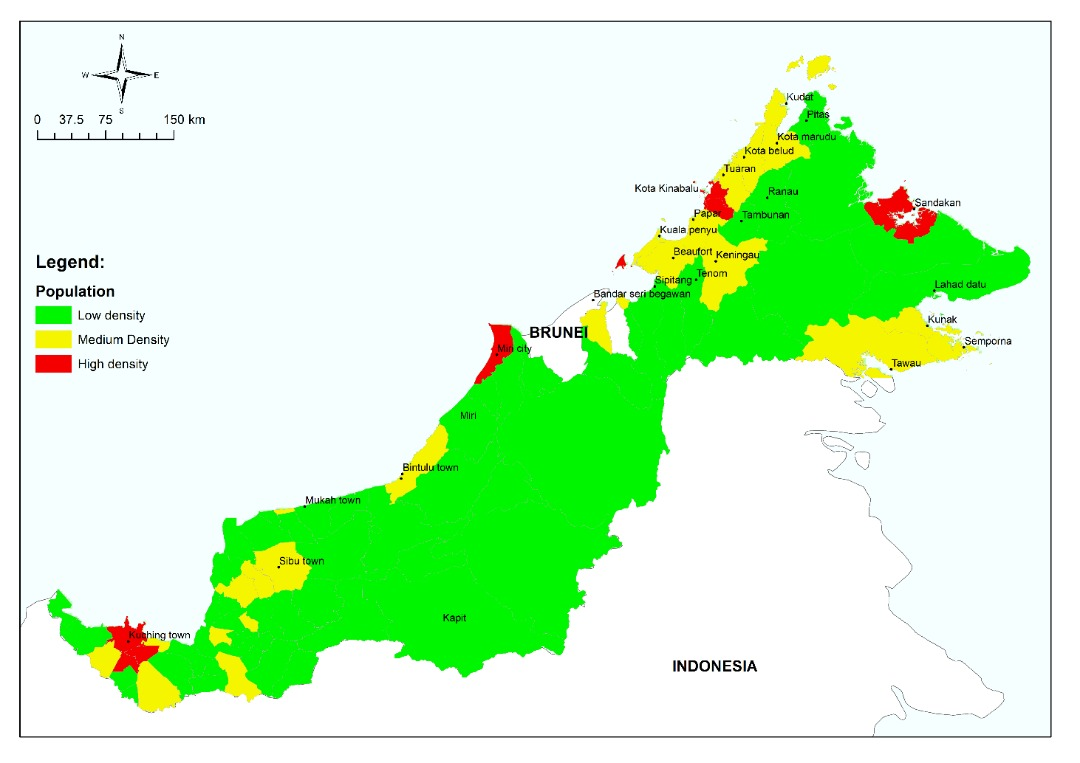

Supplement: Supplementary file 1 — Additional file 1. Population density according to district in West and East Malaysia. [file 40545_2021_308_MOESM1_ESM.docx]
